# Supplementary material for: A diverse proteome is present and enzymatically active in metabolite extracts
Source: Nat Commun. 2024 Jul 10;15:5796. doi: 10.1038/s41467-024-50128-z (PMC11237058; doi:10.1038/s41467-024-50128-z)
Supplement: Supplementary file 1 — Supplementary Information [file 41467_2024_50128_MOESM1_ESM.pdf]

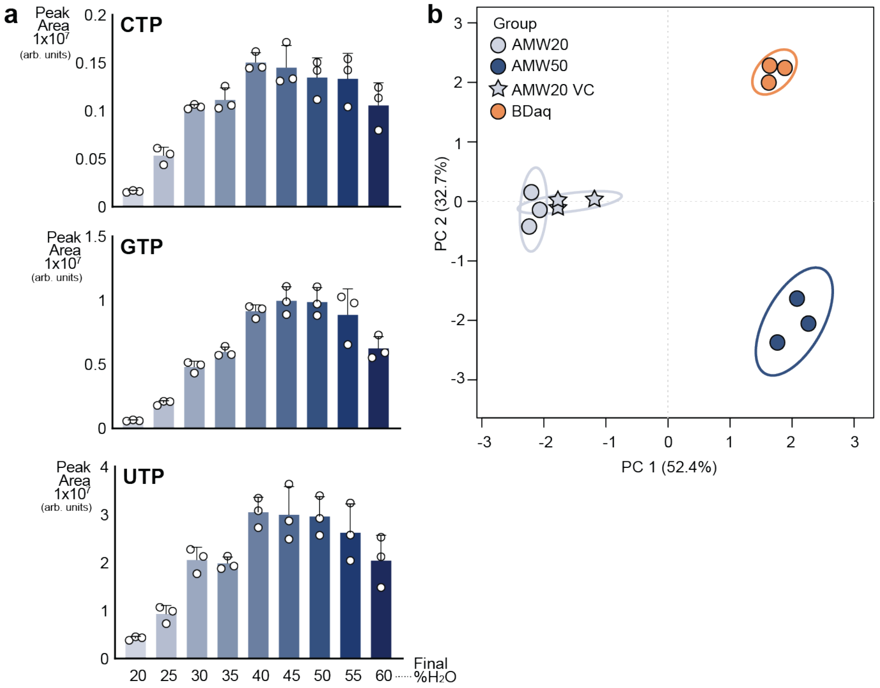

Supplementary Figure 1.

Extraction water content impacts metabolite extraction independent of extraction volume. (a) Abundance of murine liver nucleotides across extraction conditions, from AMW20 to AMW60 (mean ± SD, *n*=3 technical replicates per group). Color gradient from light blue to dark blue representing more water content in the sample. (**b**) PCA of murine liver metabolites across AMW20, AMW20 volume-control (VC; final volume equal to that of AMW50 condition, but secondary incubation was with the AMW20 solvent instead of water), AMW50, and Bligh Dyer aqueous (BDaq) extraction conditions (MetaboAnalyst 6.0, 95% confidence ellipse, *n*=3 technical replicates per group).

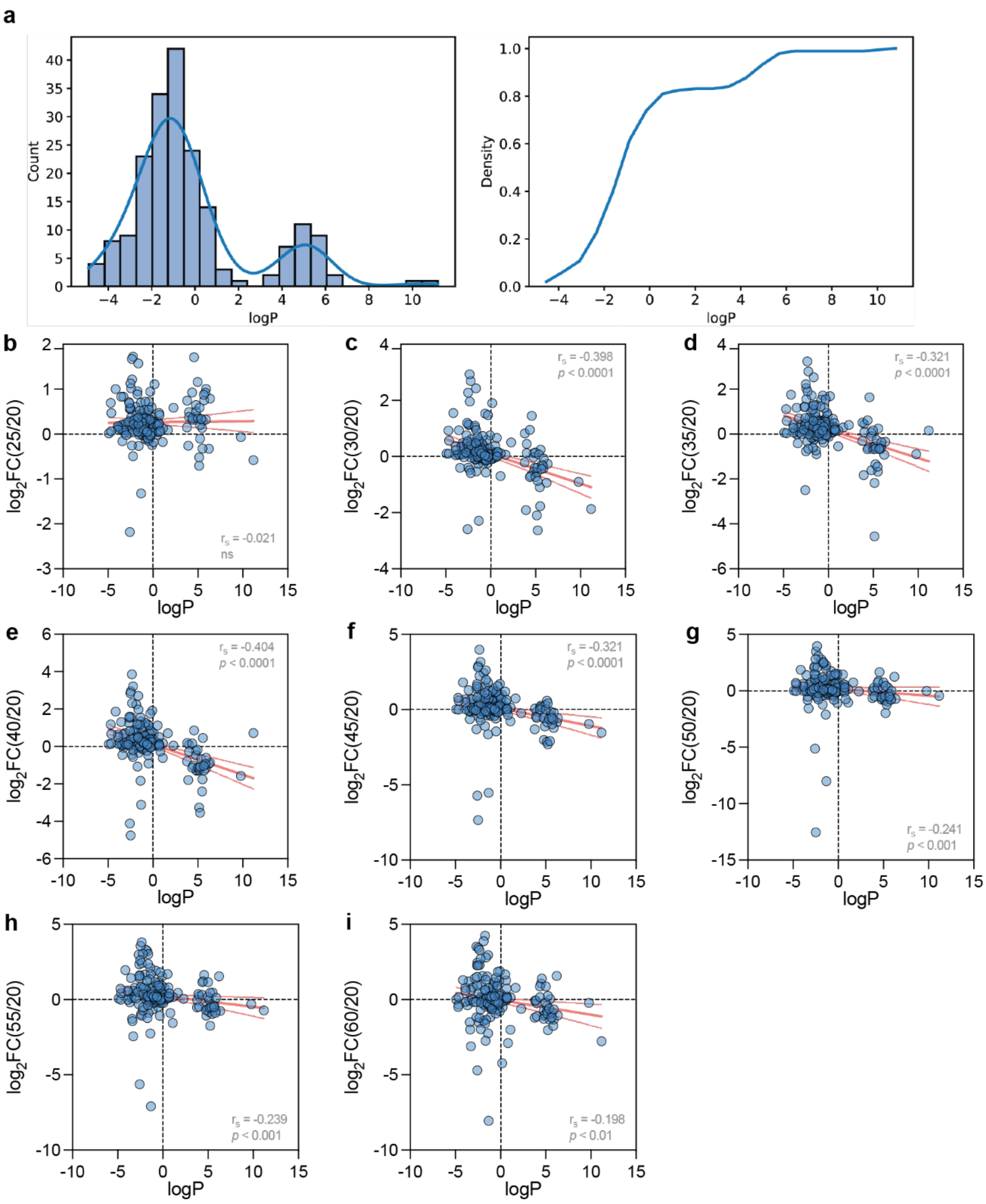

Supplementary Figure 2.

Metabolite LogP distribution. (**a**) Histogram of the octanol-water coefficient (LogP) distribution in the cheminformatics analysis. The kernel density estimate (KDE) is overlayed. The cumulative distribution function is shown to the right. The mean Log2 fold change (*n*=3 technical replicates) of metabolite levels in the (b) AMW25, (c) AMW30, (d) AMW35, (e) AMW40, (f) AMW45, (g) AMW50, (h) AMW55, (i) AMW60 extraction condition is shown relative to control (AMW20). In all cases, the Spearman correlation coefficient (r_s) was calculated for the specified Log2 fold change and LogP datasets. These values are inset in each plot together with the p-value, simple linear regression

models (red solid lines) and the 95% confidence intervals (red dashed lines) are displayed. Abbreviations: ns = not significant (determined using a 5% significance level).

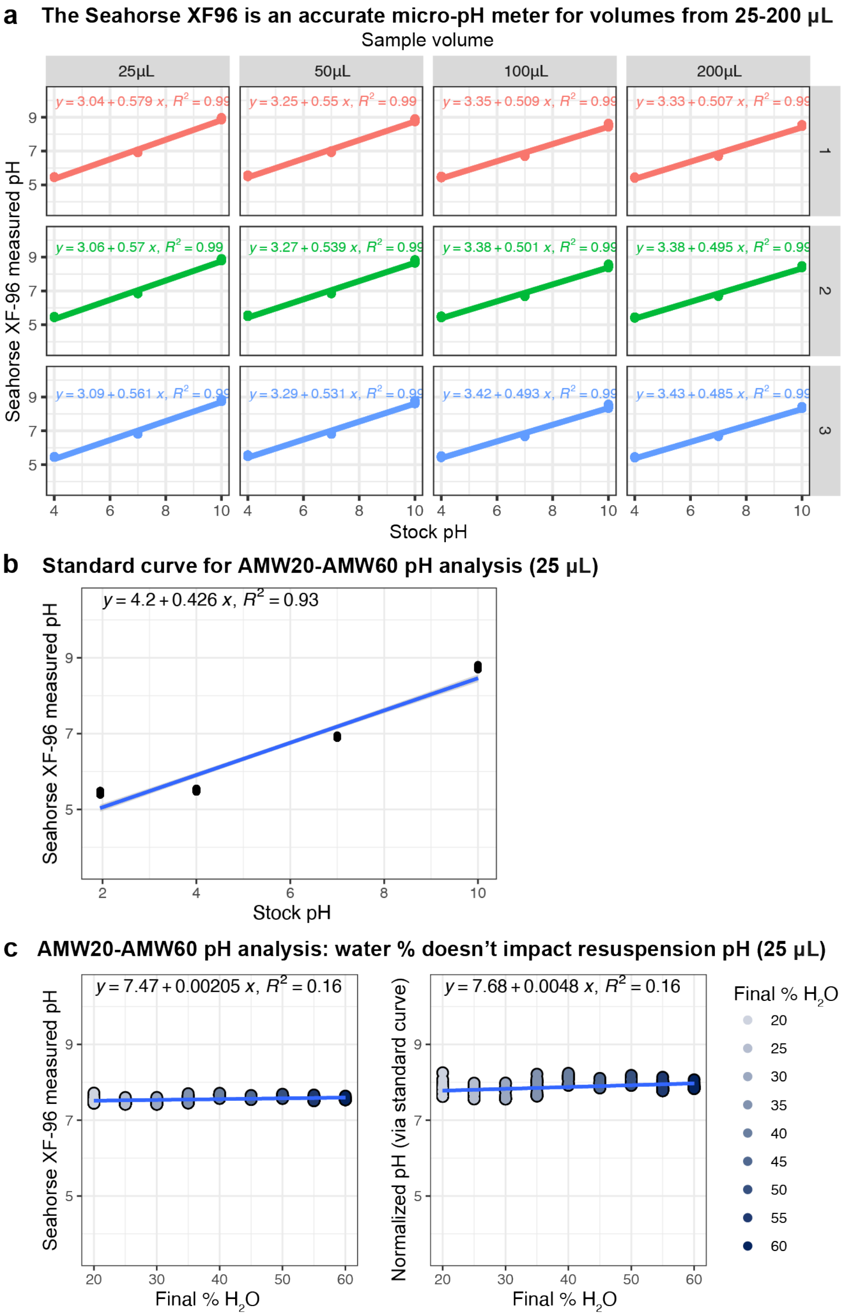

Supplementary Figure 3.

Extraction water content does not affect pH. (**a**) The Seahorse XF-96 is an accurate micro-pH meter for 25-200 µL volumes. Linear regression models (solid lines) and equations are displayed across measurements (1, 2, and 3) and sample volumes (25, 50, 100, and 200 µL). The R^2 value comparing stock pH and Seahorse F-96-measured pH values are shown. (**b**) Standard curve for the AMW20-AMW60 pH analysis. Linear regression model (solid lines) and equation are displayed. The R^2 value comparing stock pH and Seahorse F-96-measured pH values are shown. (**c**) Extraction water content (AMW20-AM60, represented by a color gradient from light to dark blue) and raw resuspension pH values do not correlate. Linear regression model (solid lines) and equation are displayed. The R^2 value comparing stock pH and Seahorse F-96-measured pH values are shown.

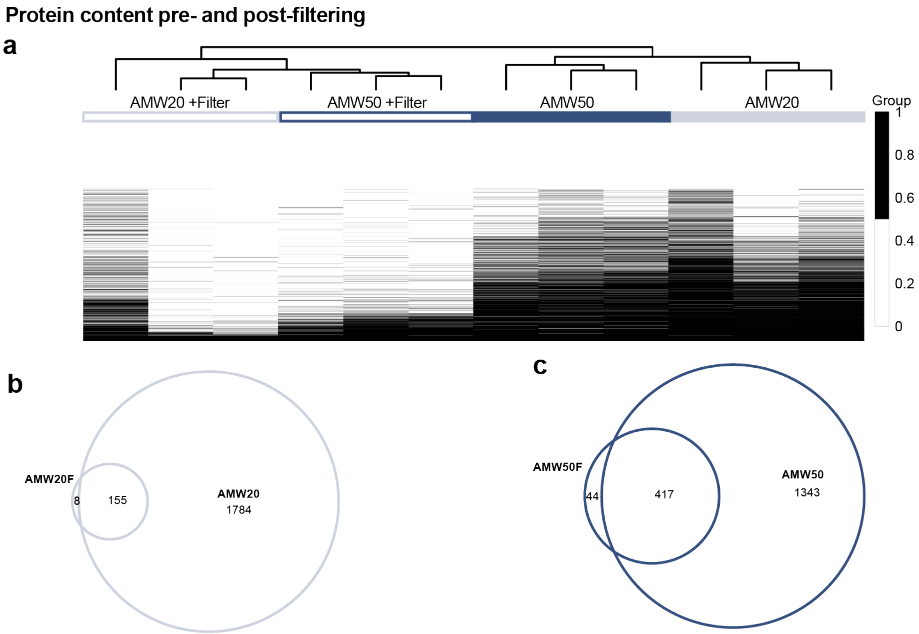

Supplementary Figure 4.

3 kDa filtration removes proteins from metabolite extracts. (**a**) Heatmap depicting relative abundance of proteins across AMW20, AMW20 + Filter (AMW20F), AMW50, and AMW50 + Filter (AMW50F) extraction conditions (**b**) Venn diagram of AMW20 and AMW20F overlapping proteins. (**c**) Venn diagram of AMW50 and AMW50F overlapping proteins.

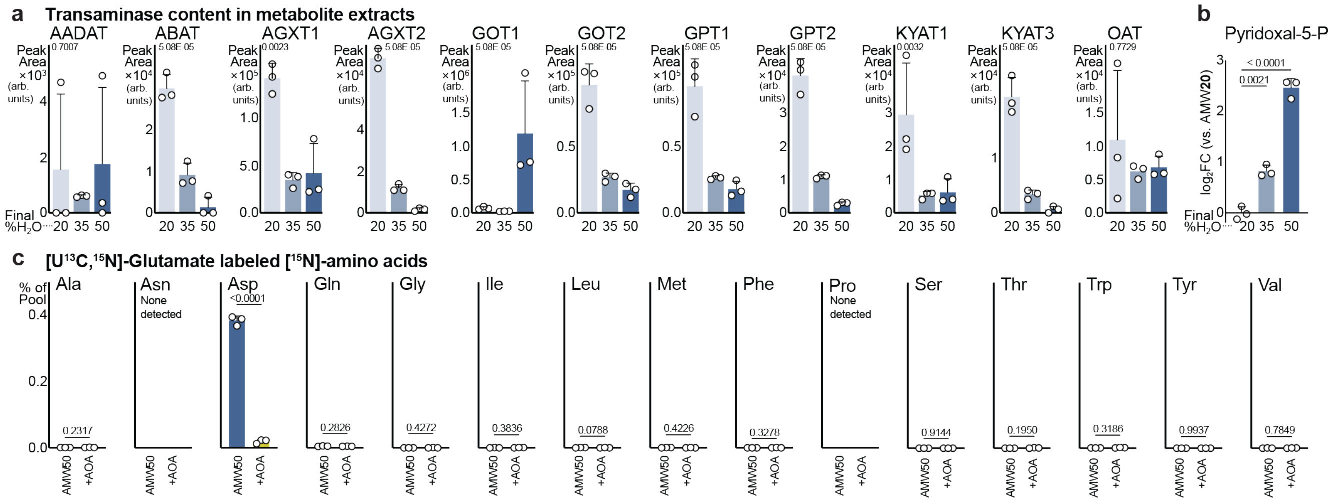

Supplementary Figure 5.

In extract transaminase abundance and [^{15}N] incorporation into amino acids from [$^{13}C_5$,^{15}N]-glutamate added during sample resuspension. (a) Abundance of murine liver transaminases in metabolite extracts from AMW20 (light blue), AMW35 (medium blue), and AMW50 (dark blue) samples. Main effect FDR value shown (mean ± SD, *n*=3 technical replicates per group). (**b**) Relative abundance (log_2FC) of pyridoxal-5-phosphate in murine liver AMW20, AMW35, and AMW50 samples. Significance calculated by Welch ANOVA (mean ± SD, *n*=3 technical replicates per group). (**c**) Relative abundance of [$^{13}C_5$,^{15}N]-glutamate -derived amino acids in murine liver in AMW50 and AMW50 + AOA extraction conditions. Significance calculated by Welch's t-test (mean ± SD, *n*=3 technical replicates per group).

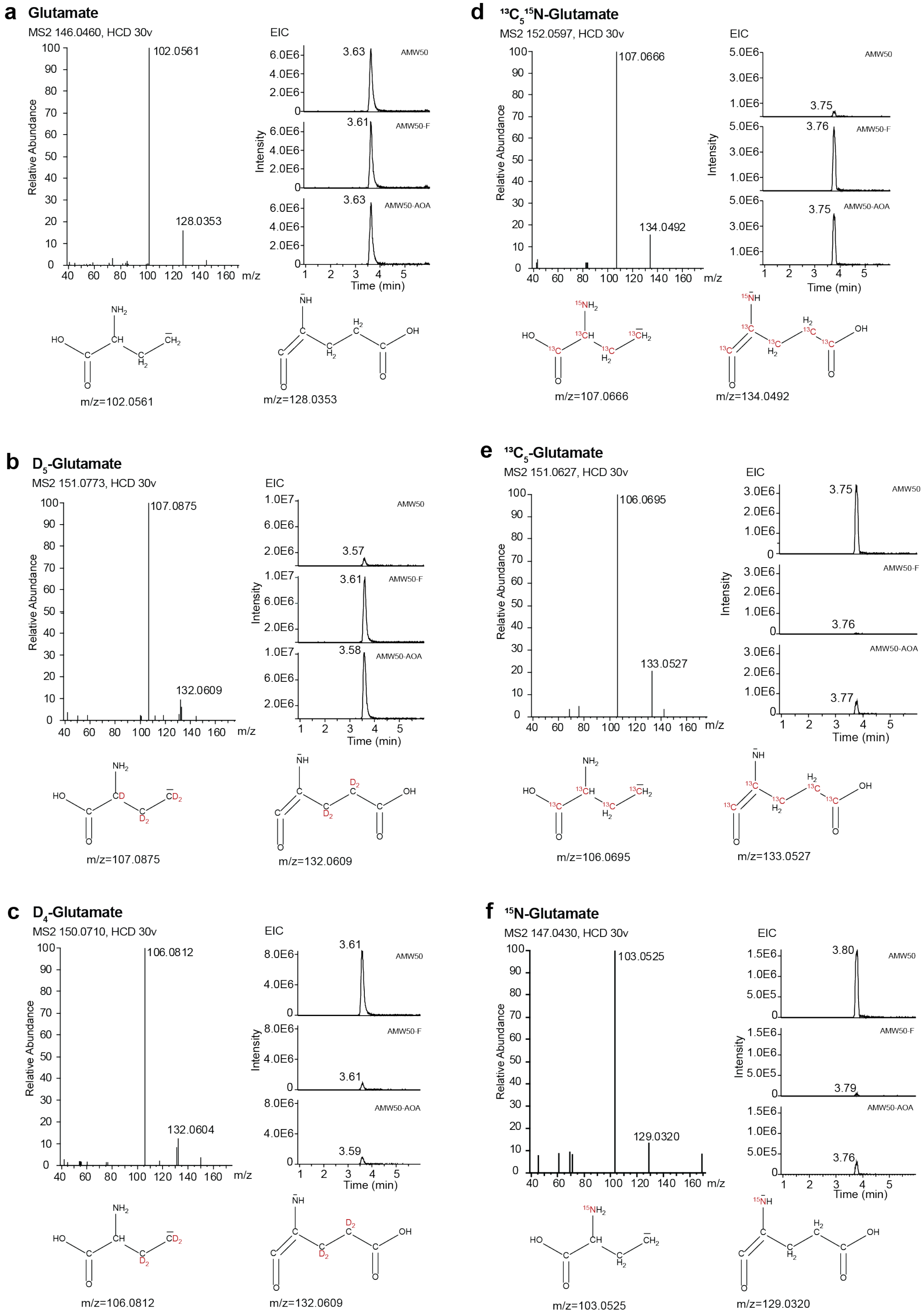

a Glutamate
MS2 146.0460, HCD 30v
102.0561
128.0353
m/z=102.0561
m/z=128.0353
b D5-Glutamate
MS2 151.0773, HCD 30v
107.0875
132.0609
m/z=107.0875
m/z=132.0609
c D4-Glutamate
MS2 150.0710, HCD 30v
106.0812
132.0604
m/z=106.0812
m/z=132.0609
d 13C5 15N-Glutamate
MS2 152.0597, HCD 30v
107.0666
134.0492
m/z=107.0666
m/z=134.0492
e 13C5-Glutamate
MS2 151.0627, HCD 30v
106.0695
133.0527
m/z=106.0695
m/z=133.0527
f 15N-Glutamate
MS2 147.0430, HCD 30v
103.0525
129.0320
m/z=103.0525
m/z=129.0320
EIC
Relative Abundance
Intensity
m/z
Time (min)
AMW50
AMW50-F
AMW50-AOA

Supplementary Figure 6.

MS2 and Extraction Ion Chromatograms (EIC) for select glutamate isotopologues in AMW50, AMW50+F, and AMW50+AOA conditions. All MS2 spectra were collected with an HCD of 30v. Location of heavy atoms are labeled in red within the associated fragmentation schematics. +F represents samples which have been filtered and +AOA is representative of samples which have been treated with the transaminase inhibitor aminooxyacetic acid. (**a**) MS2 and EIC for m/z 146.0460, Glutamate. EIC shows roughly equal abundance across sample groups (**b**) MS2 and EIC for m/z 151.0773, D_5-Glutamate. A depletion of D_5-glutamate is seen in AMW50. (**c**) MS2 and EIC for m/z 150.0710, D_4-Glutamate. Presence of the 132.0609 peak as also seen in b. Note the formation of a C=C double bond on the α-amino carbon that displaces the hydrogen at this location in the 132.0609 fragment, which present in both D4 and D5 labeled glutamate and confirms the α-amino carbon and the location of deuterium loss in **Figure 4 a-c**. An increase in D_4-glutamate is observed in AMW50 relative to other conditions. (**d**) MS2 and EIC for m/z 152.0597 [$^{13}C_5$,^{15}N]-glutamate. Depletion is observed in AMW50 (**e**) MS2 and EIC for m/z 151.0627, $^{13}C_5$-glutamate. A strong increase can be seen in the EIC for AMW50 (**f**) MS2 and EIC for m/z 147.0430, ^{15}N-glutamate. An increase is observed in AMW50.

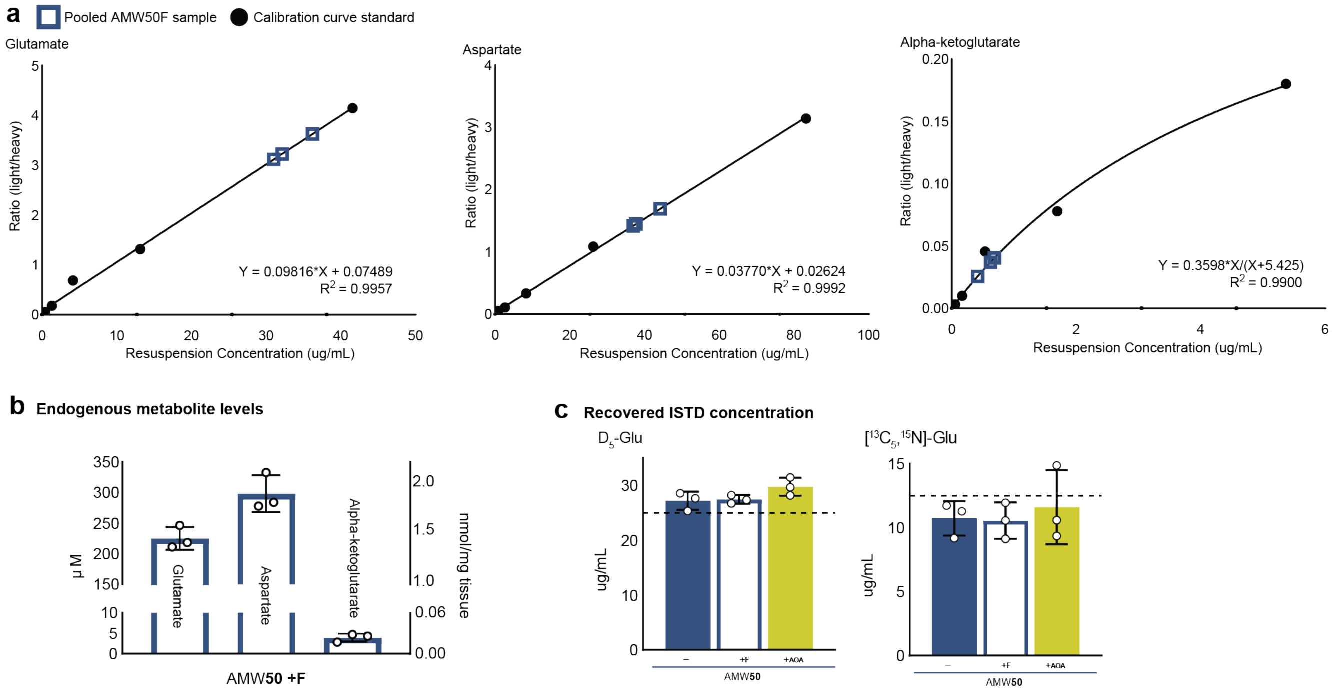

Supplementary Figure 7.

Quantitative analysis of glutamate, aspartate, and alpha-ketoglutarate. Quantitative LCMS calibration curves **(a)** were created as a ratio of unlabeled compound to spiked ^{13}C-labeled compound in a series of standards, black dots. Regression lines were calculated in Graphpad Prism, and (**b**) endogenous levels in AMW50F (filtered) samples (*n*=3 technical replicates per group) were calculated both at the resuspension concentration and corrected to the starting mg of tissue equivalents. (**c**) 25 µg per mL of D_5-Glu and 12.5 µg per mL of [$^{13}C_5{}^{15}N$]-Glu, were added as internal standards to their respective experiments, as indicated by dashed lines. The calculated concentration after accounting for any post-resuspension interconversion is shown for AMW50 (dark blue) AMW50 filtered (blue outline) and AMW50 + aminooxyacetic acid inhibitor (yellow). (mean ± SD, *n*=3 technical replicates per group).

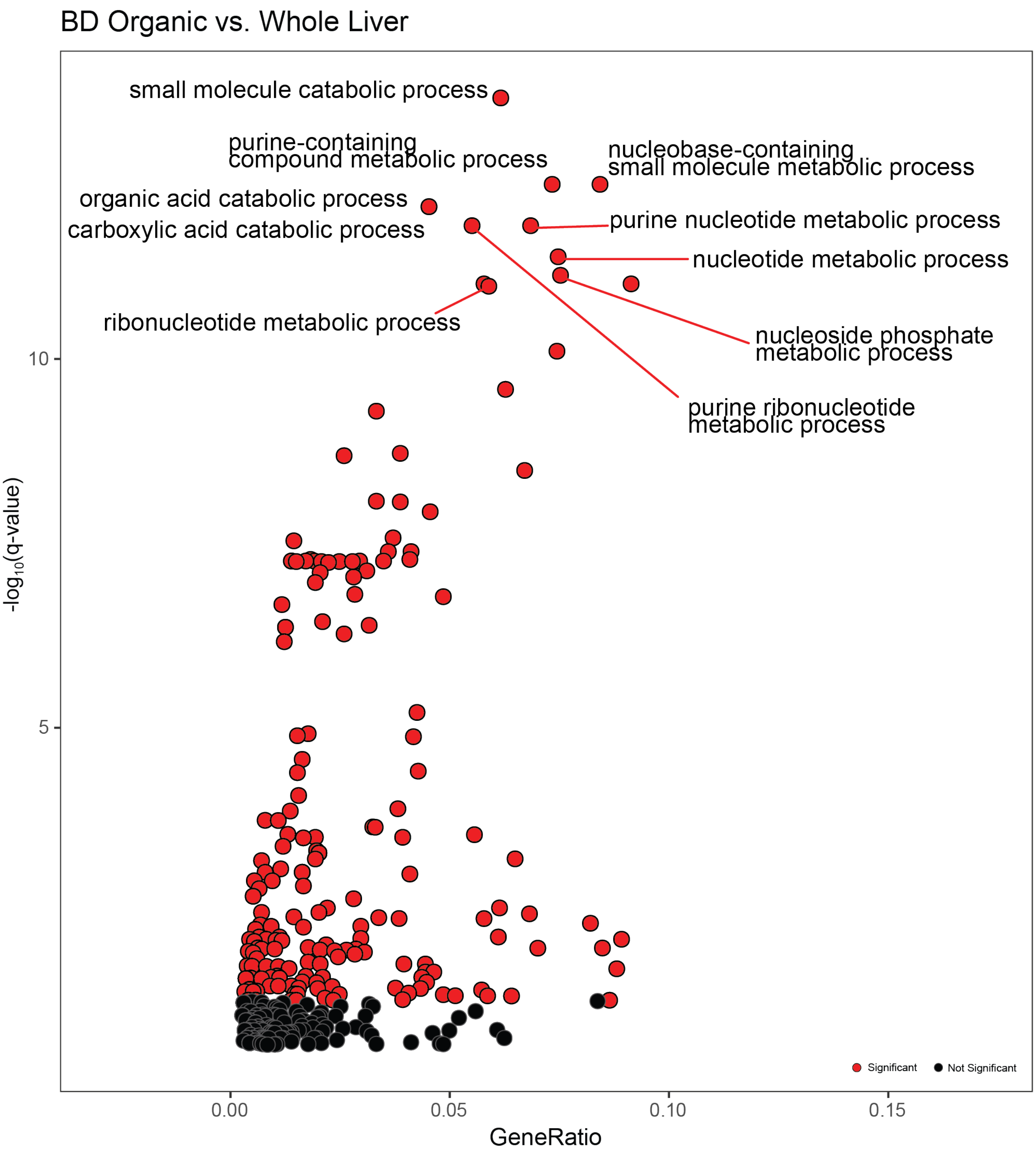

Supplementary Figure 8.

GSEA (biological processes) of Bligh Dyer-organic protein enrichment *versus* whole liver. The top 10 pathways (based on FDR q-values) are labelled. Points are colored by significance ($p<0.05$).

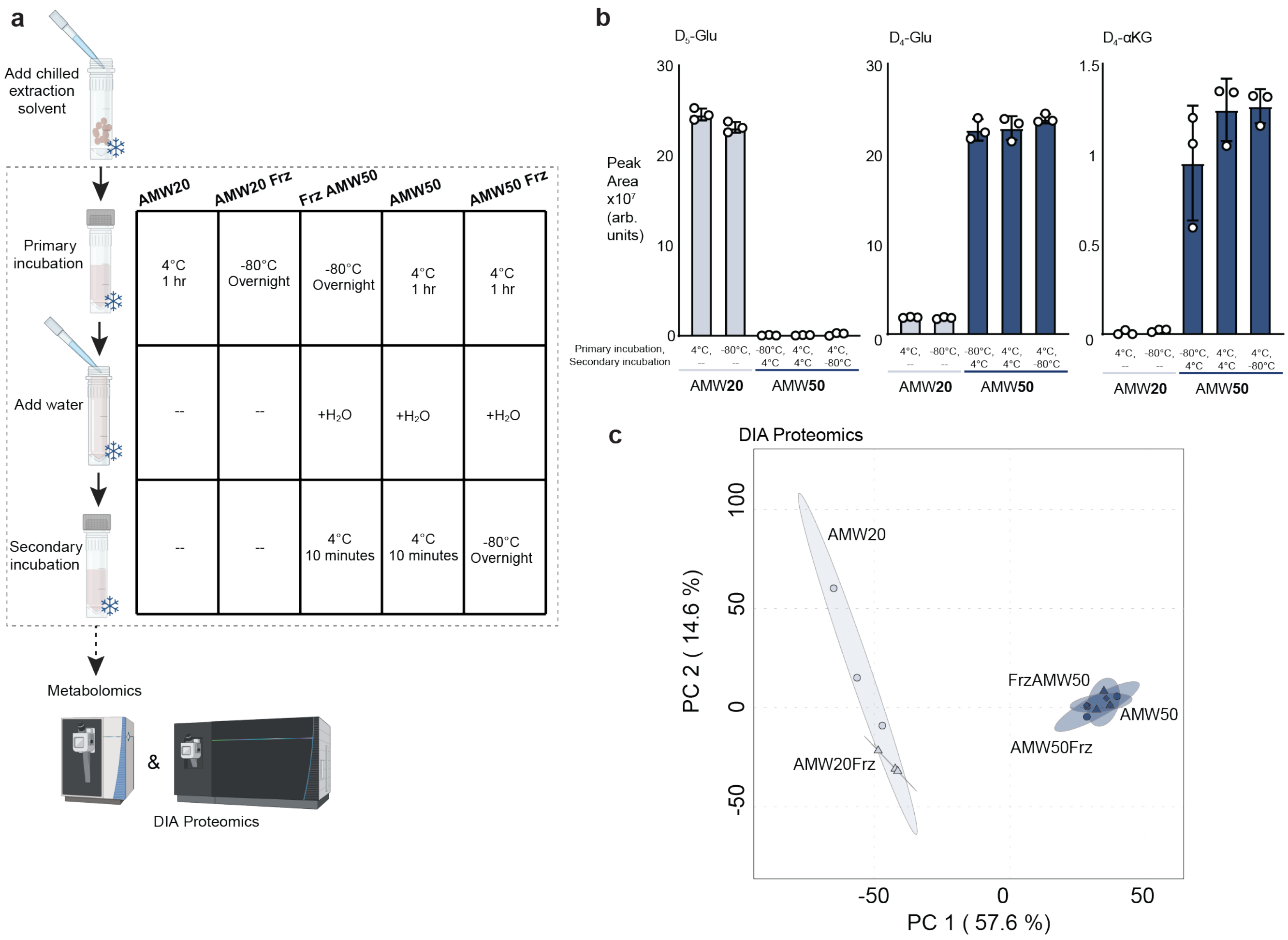

Supplementary Figure 9.

–80˚C incubation and freeze thaw cycling does not facilitate protein removal or prevent enzymatic activity. (**a**) schematic depicting the 5 conditions samples were processed under. Primary incubation was at 4 ºC for 1 hr, or –80 ºC overnight. A subset of samples which had water addition were secondarily incubated at 4 ºC for 10 min, or –80 ºC overnight. All samples were analyzed by LCMS metabolomics and quantitative proteomics. (**b**) Abundance of D$_5$-glutamate, D$_4$-glutamate, and D$_4$-alpha-ketoglutarate across extraction conditions. Dark blue bars indicate samples with added water content (**c**) PCA of proteins across AMW20, AMW20 Frz, Frz AMW50, AMW50, and AMW50 Frz extraction conditions (MetaboAnalyst 6.0, 95% confidence ellipse, *n*=3 technical replicates per group).

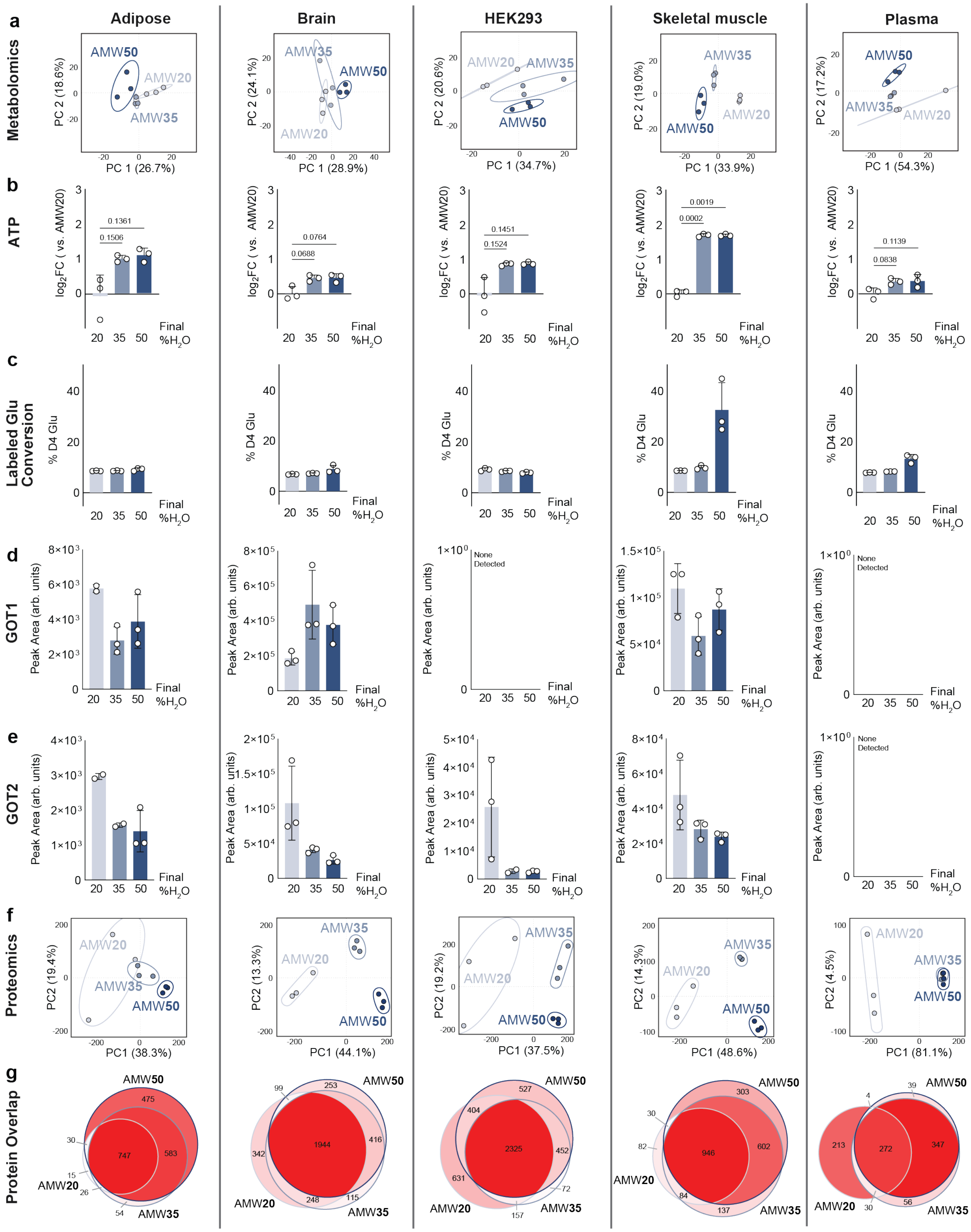

a
b
c
d
e
f
g
Adipose
Brain
HEK293
Skeletal muscle
Plasma
Metabolomics
ATP
Labeled Glu Conversion
GOT1
GOT2
Proteomics
Protein Overlap
AMW20
AMW35
AMW50
PC 1 (26.7%)
PC 2 (18.6%)
PC 1 (28.9%)
PC 2 (24.1%)
PC 1 (34.7%)
PC 2 (20.6%)
PC 1 (33.9%)
PC 2 (19.0%)
PC 1 (54.3%)
PC 2 (17.2%)
log2FC (vs. AMW20)
0.1361
0.1506
0.0764
0.0688
0.1451
0.1524
0.0019
0.0002
0.1139
0.0838
Final %H2O
% D4 Glu
Peak Area (arb. units)
None Detected
PC1 (38.3%)
PC2 (19.4%)
PC1 (44.1%)
PC2 (13.3%)
PC1 (37.5%)
PC2 (19.2%)
PC1 (48.6%)
PC2 (14.3%)
PC1 (81.1%)
PC2 (4.5%)
475
747
583
30
15
26
54
99
253
1944
416
342
248
115
527
404
2325
452
631
72
157
303
30
82
946
602
84
137
4
39
213
272
347
30
56

Supplementary Figure 10.

Multi-matrix assessment of water concentration dependence on protein content, ATP, D_5 Glu to D_4 Glu conversion. (**a**) PCA of AMW20, AMW35, and AMW50 metabolites across matrices. Matrices include murine liver, adipose, brain, muscle, and plasma, and human HEK293 cells (95% confidence ellipse, *n*=3 technical replicates per group) (**b**) Log2 fold change in ATP signal at 3 final water concentrations *versus* the average of AMW20 for adipose, brain, HEK293 cells, skeletal muscle, and plasma matrices. Significance calculated by Welch's ANOVA (mean ± SD, *n*=3 technical replicates per group). (**c**) Percentage of D_4-glutamate as a portion of the total labeled glutamate pool (D_4 Glu + D_5 Glu signal) for adipose, brain, HEK293 cells, skeletal muscle, and plasma matrices in AMW20, AMW35 and AMW50 extracts (mean ± SD, *n*= 3 technical replicates per group) (**d**) GOT1 and (**e**) GOT 2 protein detection as a function of water content in skeletal muscle metabolomics extracts. Relative abundance of GOT1 and GOT2 proteins in skeletal muscle as a function of water content (mean ± SD, *n*= 3 technical replicates per group). (**f**) PCA of AMW20, AMW35, and AMW50 proteins across matrices. Matrices include murine liver, adipose, brain, muscle, and plasma, and human HEK293 cells (95% confidence ellipse, *n*=3 technical replicates per group). (**g**) Venn Diagram of overlapping proteins identified in AMW20, AMW35, and AMW50 extraction conditions for adipose, brain, HEK293 cells, skeletal muscle, and plasma matrices.

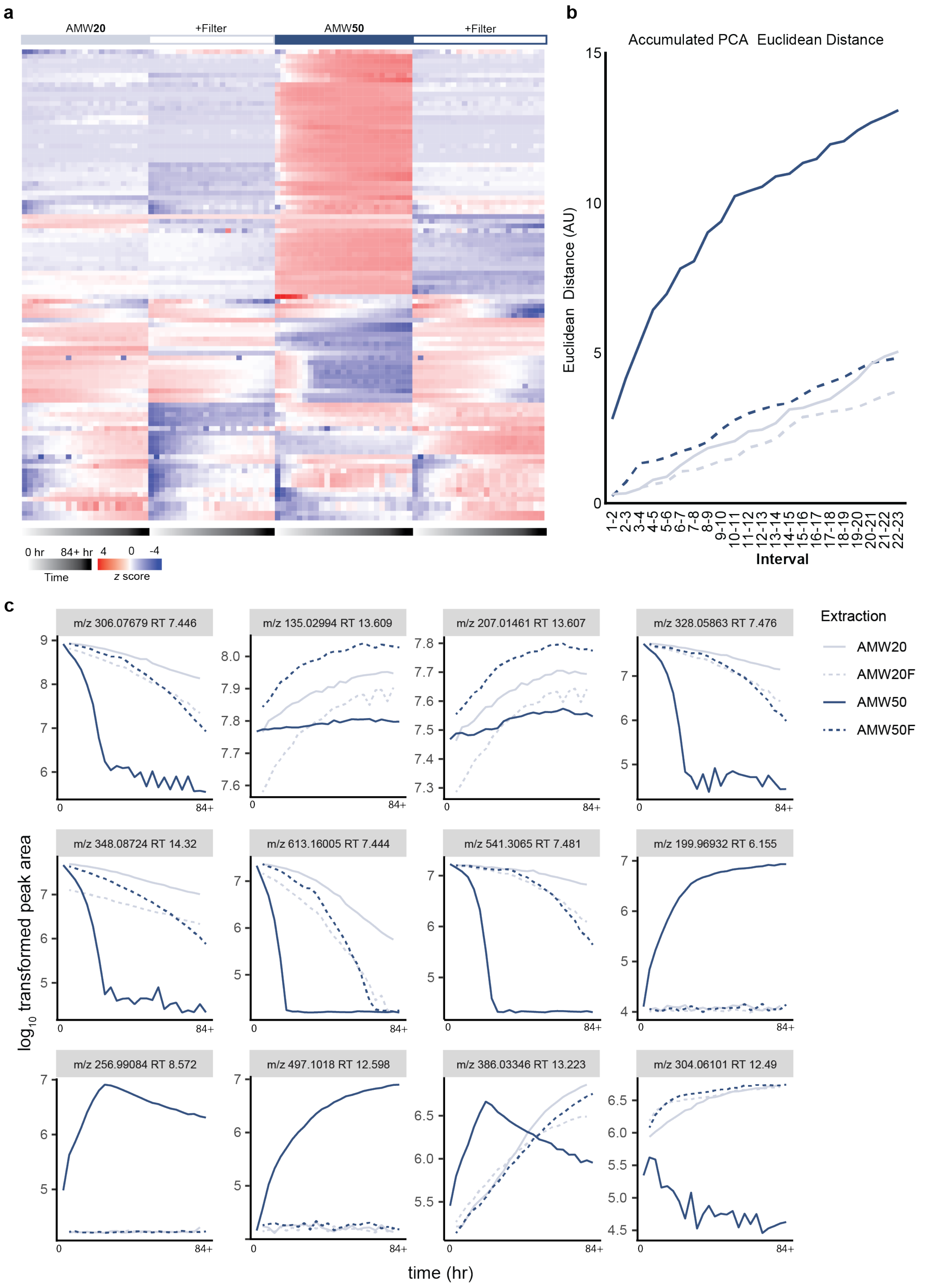
a
AMW20
+Filter
AMW50
+Filter
0 hr
84+ hr
Time
4
0
-4
z score
b
Accumulated PCA Euclidean Distance
Euclidean Distance (AU)
Interval
c
m/z 306.07679 RT 7.446
m/z 135.02994 RT 13.609
m/z 207.01461 RT 13.607
m/z 328.05863 RT 7.476
m/z 348.08724 RT 14.32
m/z 613.16005 RT 7.444
m/z 541.3065 RT 7.481
m/z 199.96932 RT 6.155
m/z 256.99084 RT 8.572
m/z 497.1018 RT 12.598
m/z 386.03346 RT 13.223
m/z 304.06101 RT 12.49
Extraction
AMW20
AMW20F
AMW50
AMW50F
$\log_{10}$ transformed peak area
time (hr)

Supplmentary Figure 11.

Time-dependent effects of extraction conditions on untargeted metabolomics. **(a)** heatmap of top 100 time-changing features, defined by non-zero slope in the generalized additive model ($p<0.05$). **(b)** Accumulated Eclidean distance calculated as the accumulated sum of the distance between sequential injections in the PCA scores plot within each group in Figure 6b. **(c)** line plots of log10 transformed peak areas from the features with the top 10 features with the lowest p-values from the generalized additive model ($p<0.05$). F indicates filtered samples.

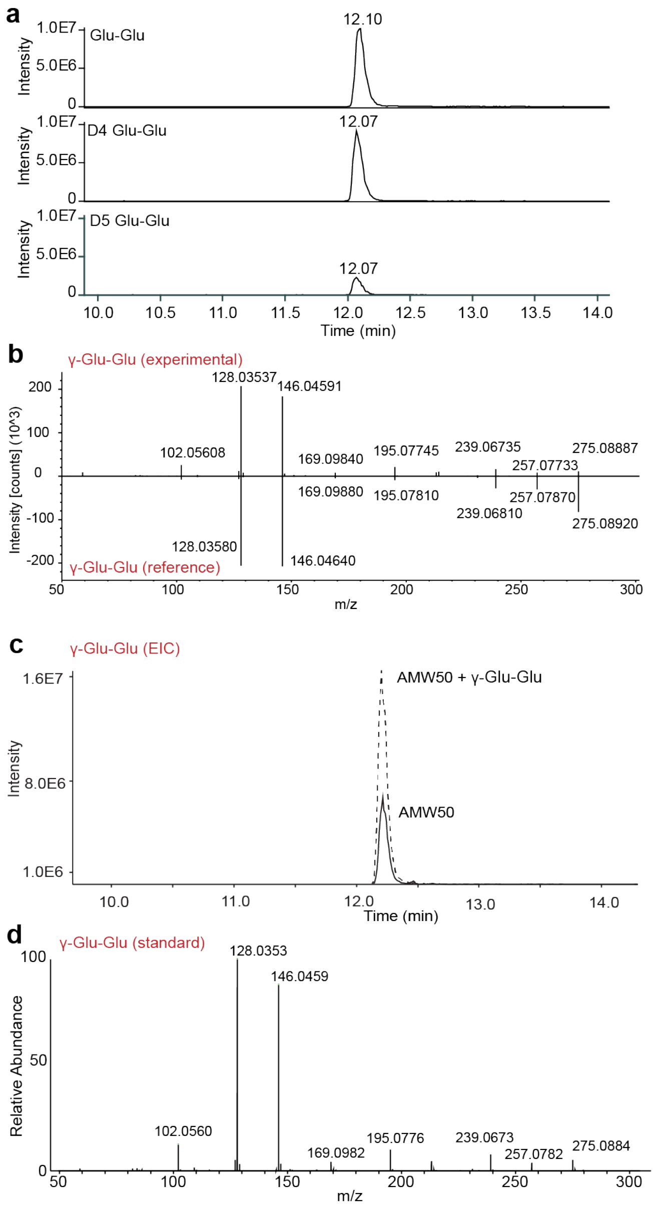

Supplementary Figure 12.

Identification of unknown feature 279.1137m/z@12.066min as D_4 labelled γ-glutamyl-glutamate. (**a**) Representative chromatographic peak alignment of extracted ion chromatograms (± 5 ppm) for m/z = 275.0886 (Glu-Glu), 279.1137 (D_4 Glu-Glu), and 280.1199 (D_5 Glu-Glu) collected from AMW50 samples. Note, deuterated compounds tend to elute slightly earlier than unlabeled cognates, explaining the RT shift from 12.1 to 12.07 min and supporting the identity of these compounds and deuterated isotopologues. (**b**) Mirror plot of data-dependent MS2 spectra from the precursor ion 275.0886@12.1min collected from AMW50 samples (top) and alignment with a library (bottom; NIST HRMS 2020 library spectral match using Compound Discoverer v3.3) spectra from a neat chemical standard (bottom). (**c**) Extracted ion chromatogram an AMW50 sample and an AMW50 sample spiked with 10µg per mL standard and **(d)** MS2 spectra of a neat analytical standard (Thermo Fisher, 228812500) confirm the identity of the unknown 279.1137m/z@12.066min as γ-glutamyl-glutamate.

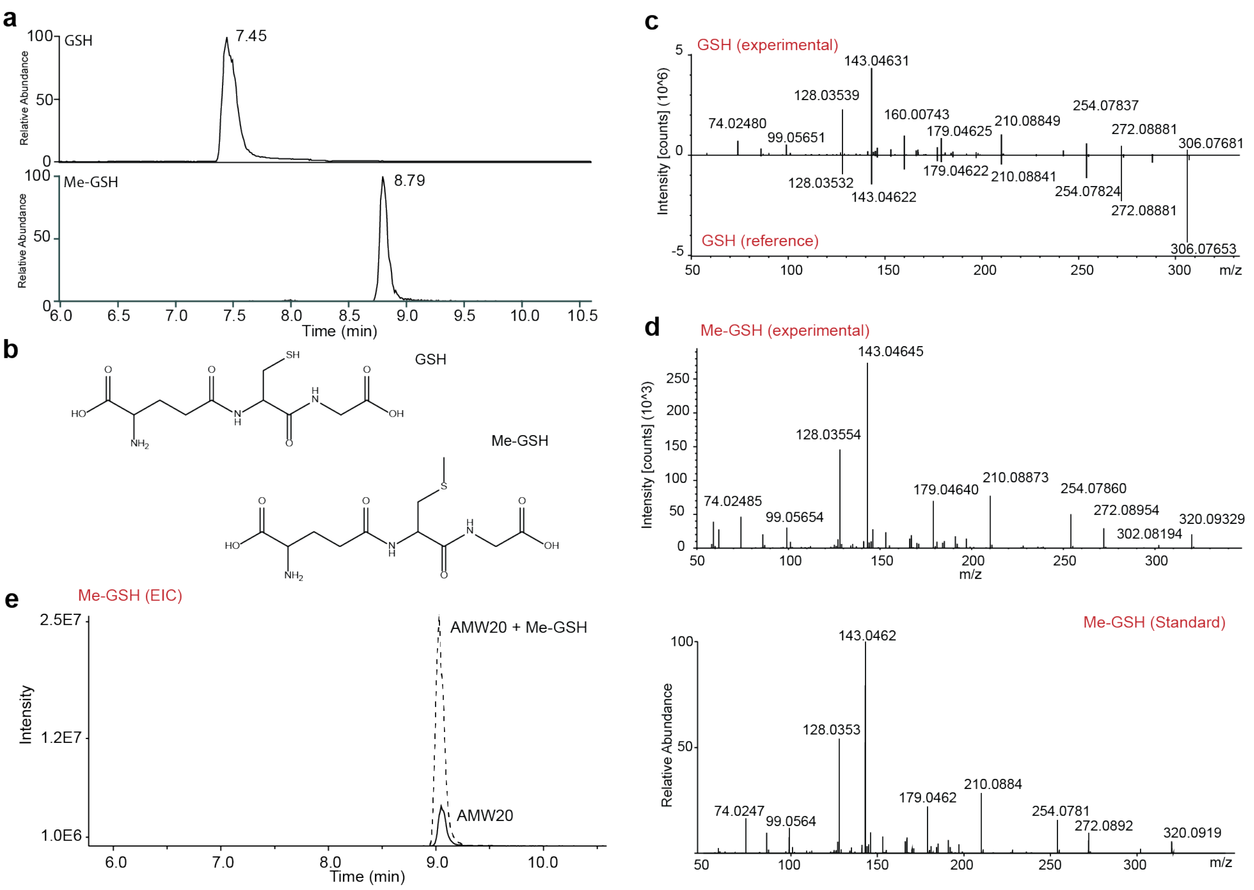

Supplementary Figure 13.

Identification of unknown feature 320.0924m/z@8.79min as S-methylglutathione. (**a**) Representative extracted ion chromatograms of reduced glutathione (top; m/z = 306.0765) and the unknown of interest (bottom; m/z = 320.0924) from an AMW50 sample. This demonstrates that these are distinct compounds and not in-source adducts/fragments of each other. (**b**) Chemical structures of GSH and S-methyl-glutathione. (**c**) Mirror plot of experimental ddMS2 spectra of glutathione (top; m/z = 306.0765@7.45 min) alignment with a spectral library (bottom; mzCloud library spectral match using Compound Discoverer v3.3). (**d**) Experimental ddMS2 spectra of m/z = 320.0924@8.8 min demonstrate strong MS2 spectral alignment with GSH, differing only in parent ion m/z, suggesting strong structural relation between the two compounds. S-methyl-glutathione MS2 spectra are not present in available databases. (**e**) Extracted ion chromatogram and MS2 spectra of a neat analytical standard (Sigma, M4139) confirm the identity of the unknown 320.0924m/z@8.79min as S-methyl-glutathione.

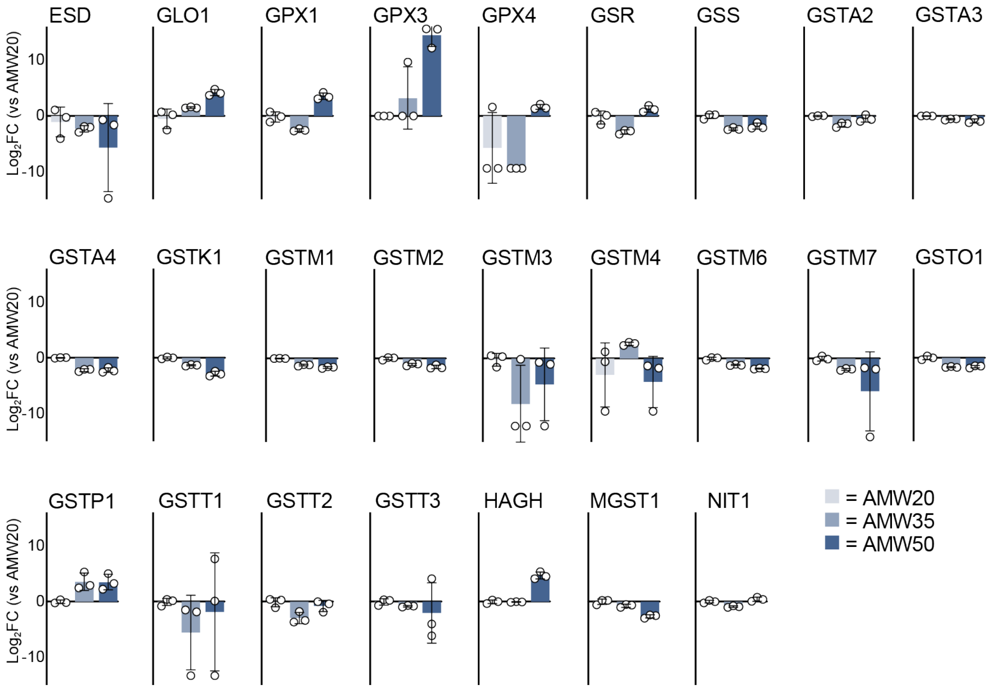

Supplementary Figure 14.

Glutathione metabolism related proteins are present in murine liver metabolite extracts. Relative abundance of murine liver glutathione-related proteins in AMW20, AMW35, and AMW50 samples (mean ± SD, *n*=3 technical replicates per group). Note, all proteins shown are present in metabolite extracts regardless of differences in abundances induced by extract water content.

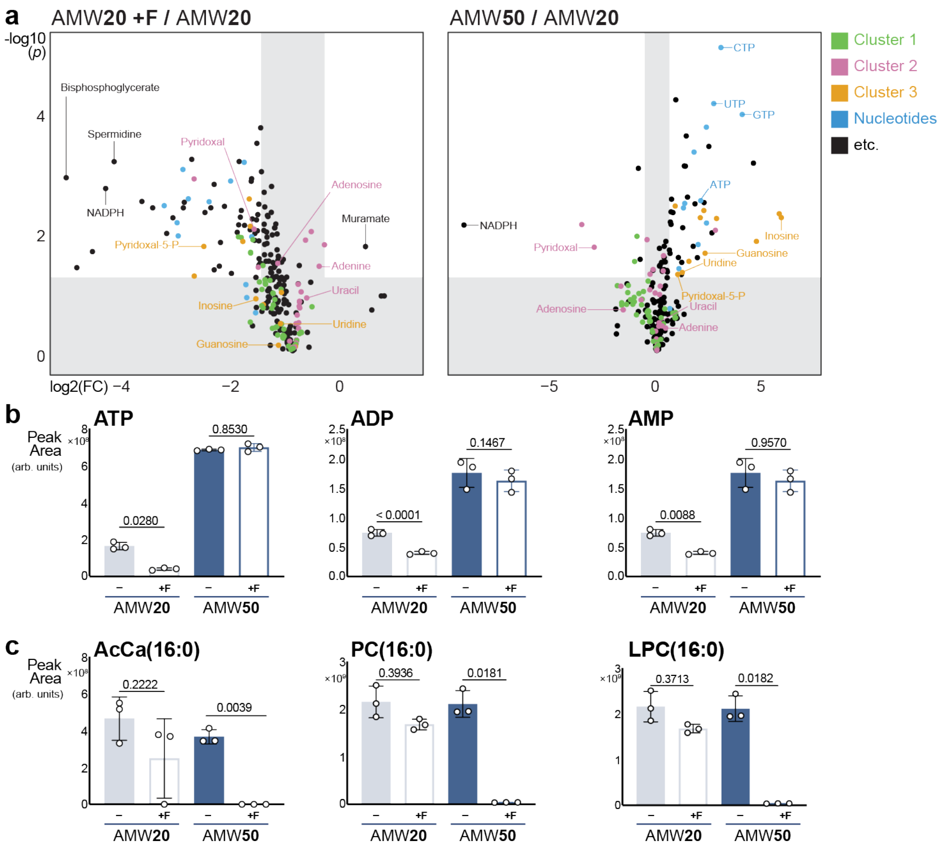

Supplementary Figure 15.

Metabolic effects of filtration in AMW20 and AMW50 conditions. (**a**) Volcano plots depicting relative abundance of murine liver metabolites between AMW20 +F and AMW20 and AMW50 and AMW20 conditions. All metabolites are shown, metabolites are colored based on cluster and metabolite class. Significance was calculated by t-test using MetaboAnalyst 6.0. (**b**) Abundance of ATP, ADP, and AMP in murine liver across AMW20, AMW20F, AMW50, and AMW50F extraction conditions. Significance calculated by Welch's ANOVA (mean ± SD, *n*=3 technical replicates per group). (**c**) Abundance of AcCa(16:0), PC(16:0), LPC(16:0) in murine liver across AMW20, AMW20F, AMW50, and AMW50F extraction conditions. Significance calculated by Welch ANOVA (mean ± SD, *n*=3 technical replicates per group).

REAGENT or RESOURCE	SOURCE	IDENTIFIER
Chemicals and reagents		
1X PBS	Gibco	10010023
Acetic Acid, Optima™ LC/MS	Fisher Chemical	A11350
Acetonitrile, Optima™ LC/MS Grade	Fisher Chemical	A955-4
Aminooxyacetic Acid	Cayman Chemical	28298
Ammonium acetate (LiChropur™)	Millipore Sigma	73594-100G-F
Chloroform, LiChrosolv	Millipore Sigma	1024441000
DMEM, no phenol red	Gibco	21063029
EDTA	VARI Media Lab	100295
Fetal bovine serum	VWR	1300-500
Formic Acid, 99.0+%, Optima™ LC/MS Grade	Fisher Chemical	A11710X1-AMP
InfinityLab deactivator additive (Medronic acid)	Agilent Technologies	5191-4506
L-Glutamic acid ($^{13}C_5$, 99%; ^{15}N, 99%)	Cambridge Isotope Laboratories	CNLM-554-H-0.25
L-Glutamic Acid (2,3,3,4,4-D_5, 97-98%)	Cambridge Isotope Laboratories	DLM-556
L-Tryptophan (D_8, 97-98%)	Cambridge Isotope Laboratories	DLM-6903-0.25
Methanol, Optima™ LC/MS Grade	Fisher Chemical	A456-4
NaCl	Gibco	S8776
Penicillin-streptomycin	Gibco	15070063
Tributylamine	Millipore Sigma	90780-100ML
Water with 0.1% formic acid, Optima™ LC/MS Grade	Fisher Chemical (via Block Scientific)	LS118-1
Water with 0.1% TFA, Optima™ LC/MS Grade	Fisher Chemical (via Block Scientific)	LS119-500
Water, Optima™ LC/MS Grade	Fisher Chemical	W6-4
Supplies		
1.5 mL Eppendorf tubes	DOT Scientific	RN1700-GMT
1.5 mL Protein LoBind® Tubes	Eppendorf	30108442
1.5 mL screw cap micro tube, low protein binding	SARSTEDT	72.703.600
2.0 mL Protein LoBind® Tubes	Eppendorf	30108450
5 mL Eppendorf tubes	Eppendorf	30119401
6-well tissue culture plates	Corning	3506
96 well Clear Flat Bottom Plates	Costar (Corning)	3370
96 Well V Bottom Plates	Greiner	651201

Adhesive 96 well plate seals	Bio-Rad	MSB1001
Amicon Ultra-2 3K centrifugal filter devices	Millipore Sigma	UFC200324
Cell scrapers	Fisher	08-100-240
Ceramic homogenizer tubes	OMNI International Inc.	19-627
Certified QSertVial™ (vial with fused-in insert)	Millipore Sigma	29391-U
Certified Vial Kit, Low Adsorption (LA), 2 mL	Millipore Sigma	29651-U
epT.I.P.S.® 0.5-20 µL	Eppendorf	22492021
epT.I.P.S.® 2-200 µL	Eppendorf	22492039
epT.I.P.S.® 20-300 µL	Eppendorf	22492047
epT.I.P.S.® 50-1000 µL	Eppendorf	22492055
LC autosampler caps		6PSC9STS1R
LC autosampler vials		6PSV9-03FIVP
Critical commercial assays		
EasyPep Mini MS Sample Prep Kit	Thermo Scientific	A40006
Pierce BCA Protein Assay Kit	Thermo Scientific	23225
Experimental models (cell lines)		
Phoenix-AMPHO	ATCC	CRL-3213
Experimental models (organisms/strains)		
Mouse: C57BL/6J	The Jackson Laboratory	RRID: IMSR_JAX:000664
Software and algorithms		
Compound Discoverer 3.3 SP2	Thermo Fisher Scientific	OPTON-31061
Graphpad Prism	GraphPad Software	https://www.graphpad.com
MetaboAnalyst 5.0 and 6.0	MetaboAnalyst	https://www.metaboanalyst.ca/
Morpheus	Morpheus	https://software.broadinstitute.org/morpheus/
R studio	Posit	https://www.rstudio.com/products/rstudio/
Skyline 23.1	MacCoss Lab Software	https://skyline.ms/project/home/begin.view
Spectronaut v18	Biognosys	https://biognosys.com/resources/spectronaut-18/

Supplementary Table 1.

List of reagents and resources used in this study. Including manufacturer and catalogue number.
