# Supplementary material for: A diverse proteome is present and enzymatically active in metabolite extracts
Source: Nat Commun. 2024 Jul 10;15:5796. doi: 10.1038/s41467-024-50128-z (PMC11237058; doi:10.1038/s41467-024-50128-z)
Supplement: Supplementary file 2 — Description of Additional Supplementary Files [file 41467_2024_50128_MOESM2_ESM.pdf]

File Name: Supplementary Data 1.

Description: metabolite profiling data related to Figure 1, Figure 5, and Figure 7 in separate tabs. Metabolite names Include the method identifier delimited by an underscore (i.e. "_ip" = ion paired method, "_hilicA"= low pH amide method, and "_t3" = T3 reversed phase method).

File Name: Supplementary Data 2.

Description: quantitative DIA proteomics data from Figure 3 and Figure 5 in separate tabs.

File Name: Supplementary Data 3.

Description: curated compound lists (compound name, chemical formula, adduct, RT, RT window) for each of the three LCMS methods.
